# Deterrence, Resilience, and the Shooting Down of Flight MH17

**DOI:** 10.1007/978-94-6265-419-8_19

**Published:** 2014-12-15

**Authors:** Cees van Doorn, Theo Brinkel

**Affiliations:** 3 Faculty of Military Sciences, Netherlands Defence Academy, Breda, The Netherlands; 4 Faculty of Military Sciences, Netherlands Defence Academy, Breda, The Netherlands; Netherlands Defence Academy, Breda, The Netherlands

**Keywords:** MH-17, BUK, hybrid threats, resilience, social capital, trust, narrative

## Abstract

Russian disinformation has thus far proven to be unconvincing for most Dutch target audiences. This is the conclusion of the present chapter. Information and disinformation have become effective weapons in international politics. This is part of a development where the weapons and concepts used in deterrence strategies have moved away from the military domain toward the political, economic, humanitarian, and communicative ones. In western literature, this is called hybrid warfare. In recent literature on hybrid warfare, resilience is often considered a key theme which may boost deterrence against hybrid activities and/or lower their impact. Most research on resilience and security is focused on infrastructure and resource planning. In this chapter, however, we attempt to ascertain how the existence of resilience in society can be observed. By looking at the case of the Dutch reaction to the shooting down of flight MH17, we hope to illustrate how resilience works in deterrence to hybrid warfare. We try to establish how subversive Russian activities were taking place and what measures were taken by the Netherlands government in order to counteract them. We monitored societal resilience by looking for the presence of trust, social capital, and credible narratives in reaction to disinformation activities after a disruptive event. All these elements appeared to be present in the MH17 case. Overall, we conclude, the handling of the MH17 case has reinforced deterrence.

## Introduction

On 17 July 2014, a Russian Buk missile shot down Malaysia Airlines flight 17 (MH17) during its flight from Amsterdam to Kuala Lumpur. The disaster occurred near an Eastern Ukrainian village called Hrabove. The crash site was situated in a conflict zone where pro-Russian separatists, including the Donbass People’s Militia, were fighting regular Ukrainian troops. As a result of the crash, 298 passengers lost their lives. The victims originated from 10 different countries; most of them were Dutch (193), Malaysian (43), and Australian (27). Shortly after the shooting down of MH17, various actors began spreading messages that were meant to convince the Dutch public of the Russian allegation that Ukraine was responsible for the massacre. While these actors were mainly Russians, Ukrainians and even some Dutch nationals were also involved.^1^

The nature and intensity of disinformation campaigns such as these have widely been recognized. Information has become an effective weapon in international politics. This is part of a development where the weapons and concepts used in deterrence strategies have moved away from the military domain toward the political, economic, humanitarian, and communicative ones. In Western literature, this is called hybrid warfare. It ranges from support for populist parties and disinformation campaigns to the utilization of organized crime and individuals sympathetic to Moscow, from intelligence operations to military pressure. The general purpose is to create a political and cultural environment that serves the interests of the Russian Federation and weakens the cohesiveness of NATO and the European Union.^2^

The question is how these subversive Russian activities were taking place and what measures were taken by Western, or—to be more specific—the Netherlands government in order to counteract them. In recent literature on hybrid warfare, resilience is often considered a key theme which may boost deterrence against hybrid activities and/or lower their impact. By utilizing findings regarding flight MH17 from previous research by Cees van Doorn, we hope to illustrate how resilience works in a context of deterrence in hybrid warfare.^3^ Most research on resilience and security is focused on infrastructure and resource planning. We attempt to ascertain how the existence of societal resilience can be observed and how the findings relate to deterrence. To do so, we conducted a literature review to define the concepts of deterrence and resilience, which resulted in an analytical framework that can be used to monitor resilience in the case of flight MH17. A group of respondents was selected who were—or still are—highly involved in the case of flight MH17. They were interviewed and their comments are used as illustrations of the way the disaster was dealt with.^4^ The final course of action comprised an analysis of the publications of government research agencies and others.^5^ Voluntary digital forensics organizations, such as Bellingcat and the EEAS, repudiate disinformation by investigating narratives, sources, and channels. Bellingcat runs an international program to educate and train its volunteer network of digital forensic investigators. Web-based digital developments have enabled voluntary digital forensics organizations, as well as other individuals, to investigate and publicly debunk disinformation.^6^

## Hybrid Threats

Deterrence has been the West’s classic answer to outside threats. During the Cold War era, a war in Europe would have been so destructive that all efforts were focused on preventing such a disaster, which has traditionally been achieved by deterring the opponent (i.e., the Soviet Union) from even considering an armed attack. The possession and thus potential use of a vast array of nuclear weapons was considered a crucial element in this situation of mutually assured destruction. After the fall of the communist system, this type of conceptualization of deterrence moved to the background. Following the attacks of 9/11, deterrence was discussed mainly in relation to terrorism. The question was whether deterrence would be possible vis-à-vis terrorists for whom death is simply an entry into paradise. Authors such as Wilner argue that terrorists can indeed be deterred, if their behaviour and motivations can be manipulated by coercive, diplomatic, and ideological measures.^7^ This argument has gained relevance in the context of hybrid warfare as well.

After the Russian seizure of Crimea in 2014 and the emergence of hybrid warfare, deterrence gained renewed acumen.^8^ Hybrid warfare has been defined by Cullen as “the synchronized use of multiple instruments of power related to specific vulnerabilities across the full spectrum of societal functions to achieve synergistic effects”.^9^ The debate on hybrid warfare broadened from a predominantly military phenomenon to a strategy which includes the whole of society. In the latter sense, hybrid warfare allows for vertical escalation, whereby one type of instrument, such as the military, is intensified, or for horizontal escalation, which means that more instruments (economic, communicative) will be put to work apart from the one already utilized.

Hybrid warfare opens the possibility to use all instruments short of actual war. Information warfare, to be more specific: disinformation campaigns, are a crucial element in this type of measure. By creating events, spreading fake news, communicating alternative narratives, strategies such as these disinformation campaigns are aimed at the heart of Western societies and the morale of the population. They are a symptom of what Rupert Smith called “war amongst the people”, where the loyalty of the population is at stake.^10^ War amongst the people essentially points at the absence of a traditional battlefield where identifiable armies are supposed to do physical battle against each other. Today’s war theatres are the streets, the households, the countryside, the Internet, the convictions and fears of the people.^11^ The West must learn to contend with strategic communication, disinformation, cyberattacks, the manipulation of social unrest, and the use of unmanned drones.^12^

Several examples of information warfare have been identified. Intellectuals and think-tanks, the Russian Orthodox Church, Russian media such as RT and Sputnik news agency are used to discuss pro-Russian narratives. According to Galeotti, insurgents, terrorists, paramilitaries and criminal groups can all be deployed in Kremlin’s cause.^13^ Treverton et al. (2018) mention the targeting of the Democratic Campaign in 2015 and 2016 in the United States by cyber operations that were linked to Russian Intelligence in support of the presidential candidature of Donald Trump. Later, such groups turned to the Netherlands, Germany and France, all in support of anti-European Union parties and candidates.^14^ In its latest annual report, the Dutch Central Intelligence and Security Service (AIVD) discusses covert political influencing that is more intrusive than normal diplomacy or political lobbying. According to the AIVD, Russia is the main originator and has a strong association with the shooting down of MH17. The impact in the Netherlands has thus far been limited.^15^ The Dutch ministers of Security and Justice and Home Affairs have judged this type of interference as highly undesirable, because this way foreign state actors affect the foundations of the democratic legal order and the open society.^16^

A classic military offensive would mean that NATO member-states invoke Article Five of the Washington Treaty, whereby an attack against one is considered an attack against all. This invocation rests upon the feeling of mutual solidarity among the NATO-members. Information warfare, aimed at influencing the people and the political leaders of the member-states, can undermine this feeling. By information warfare, Moscow aims to foster dividedness among citizens and distrust towards their governments, the EU and NATO. Authoritarian populist movements in the West itself and politicians and parties as Alternative für Deutschland, Front National in France, Jobbik in Hungary and the UKIP in the United Kingdom, play into the hands of Russia.^17^ To quote Rupert Smith: “The battlefields of today are the streets, the households, the countryside, the Internet, the convictions and fears of the people.”^18^ Hybrid strategies and information warfare place new demands on the concept of deterrence.

## Deterrence and Resilience

According to NATO, deterrence is the capability to deter an opponent from taking aggressive action against members of the alliance.^19^ In the classical view, this effect is achieved in two ways: deterrence by punishment, which means both the credible threat and the actual capability to retaliate after an enemy attack, and deterrence by denial, which regards the capability to block the ambitions of the opponent where NATO members would be the victims. In both aspects, the word “capability” is essential, as capability embraces the means (e.g., military personnel and equipment, infrastructure, and economic capacities) and the political resolve to employ these means.^20^

With the emergence of the recent debate on hybrid strategies and information warfare, a third way was added to that of denial and punishment: deterrence by delegitimization. This concept is based on the idea that non-traditional opponents, such as terrorists, are politically motivated. Wilner argues that the chances of achieving their political targets diminish when the foundations of their political motivation—such as publicity, cohesion, or sympathy among the population—are delegitimized.^21^ According to Knopf this approach involves challenging terrorists’ justifications for violence, an approach that has been labelled deterrence by counter-narrative or deterrence by delegitimization.^22^ According to an information note of the Countering Hybrid Warfare project of 14 Western countries and the EU, the same logic applies to actors who use hybrid warfare strategies.^23^

In this section, we will discuss the meaning of the concept of resilience first and then its relevance to deterrence in a context of hybrid strategies and information warfare. Resilience, according to Rodin, “is the capacity of any entity—an individual, a community, an organization, or a natural system—to prepare for disruptions, to recover from shocks and stresses, and to adapt and grow from a disruptive experience”.^24^ Resilience usually concerns technical solutions and infrastructure; nevertheless, according to Rodin, resilience can also be found in attitudes, declarations, and images and observed in public debate and common values and objectives. In that respect, resilience is part of the social capital and trust in society, as well as the narratives that guide it.^25^

The general relationship between resilience and security is broadly recognised. According to Fjäder, for instance, security and resilience are both part of the current security paradigm. Security is preventive and proactive, whereas resilience is a combination of proactive and reactive measures, not directed at one particular threat but at all kinds of human, technical or natural disasters. Security is connected to territory, whereas resilience is more connected with a complex system, institutions, or a value chain. Resilience can contribute to security. A resilient society and a strong defence work as a deterrent that help to prevent an attack or an assault.^26^ But there are also more specific reasons why resilience enhances deterrence in the context of hybrid warfare.

First, because it is impossible to deter all elements of the complex and diverse scope of hybrid strategies. Total defence is not feasible. According to Coaffee and Wood, resilience is a social condition that is helpful in managing the way risks are dealt with.^27^ A resilient society will undermine and deny inimical efforts in many of the domains in which they may occur.

Second, because deterrence in hybrid warfare amounts to a test of will, Giegerich argues, resilience must be enhanced.^28^ Deterrence depends on our societies making the desired impression on the opponent through the strength of our defence posture and our political resolve. Galeotti conveyed it plainly: The Kremlin must be convinced that the costs of political warfare are higher than potential gains.^29^ Capabilities that impress the opponent, a track record of promises kept and consistency in policies all enhance deterrence, according to Gray. Furthermore, in his view, enemies play a role in that they will process the messages they receive and decide whether they will be deterred. They may recognize the strength of the defender’s defence posture but simultaneously be unimpressed by its political resolve.^30^ In a paper on the defence of the Nordic countries, Whiter indicates how they acknowledged the relevance of societal resilience. Finland, for instance, uses the term “psychological resilience”, which is defined as “the ability of individuals, communities, society, and the nation to withstand the pressures arising from crisis situations and to recover from their impacts”. Psychological resilience is seen there as a critical factor in the political determination of the Finnish population.^31^

Finally, in a context of hybrid strategies and information warfare, credibility can ultimately become the decisive weapon in defence of the West. In the view of Nicolini and Janda, in propaganda campaigns, disinformation is an often-used instrument. Veracity, consistency and respect for the truth are the exact opposite and enhance what has been described above as deterrence by delegitimization.^32^ In its search for ways to deter hybrid threats, the European External Action Service (EEAS) of the EU stressed the importance of resilience. In a Food for Thought Paper for the EEAS, good governance and human rights and freedoms, as well as rule of law, fighting corruption, and a better system for funding political parties, were mentioned as “key ingredients in the fight against hybrid attack”.^33^ As was said above: total defence is not feasible. Nor is it desirable.

NATO has recognised the importance of resilience in deterring hybrid warfare by a renewed appreciation of Article Three of the Washington Treaty, the founding agreement of the North Atlantic alliance. Article Three provides that the allies “separately and jointly, by means of continuous and effective self-help and mutual aid, will maintain and develop their individual and collective capacity to resist armed attack.”

## Monitoring Resilience

In the previous section, we argued that resilience enhances deterrence in several ways. In this section we consider the way resilience plays a role in the Russian information campaigns regarding the shooting down of flight MH17. We have taken the 2014 shooting down of flight MH17 as a case for our study because it has been an important disruptive experience, first for the families and friends of the victims. The attack has also affected Dutch society. It brought war closer to the people in the Netherlands; we respect that. What interests us here is the observation that the Putin regime immediately used this shooting down to come up with narratives which would foster uncertainty and distrust in the West in general and the Netherlands in particular.

In monitoring the presence of resilience in information warfare we need a workable set of building blocks that can serve as markers to help observe its presence. Societal resilience as such is not directly visible or measurable. But by looking at the aims of information warfare and its objectives it is possible to identify markers of a resilient society. As was stated above, by information warfare, Moscow aims to foster dividedness among citizens and distrust towards their governments, the EU and NATO. The opposite of distrust of citizens towards their governments is trust; the opposite of dividedness is social capital; the opposite of disinformation is a credible narrative. These markers have been selected on the basis of the criteria developed by the Stockholm Resilience Centre, Noordegraaf et al. (2018) and Versteegden.^34^

Trust means building trust in the nation and its institutions and combat distrust among citizens towards their governments, the EU and NATO. Trust is the willingness of citizens to believe in the authorities’ ability to manage a crisis in the face of uncertainty, whereby citizens believe that the government will abide by ordinary ethical rules (e.g., telling the truth).^35^ Social capital, in turn, relates to the bonds that hold people together. According to Durodié resilience has to do with the idea of who we are and where as a society we are heading. Feelings of social solidarity and self-sacrifice in society are important elements against dividedness.^36^

The use of a credible narrative, truth and transparency, can counter disinformation. The function of a narrative is to bring order in a world that is perceived as chaotic and unpredictable and serve as a framework. A strong narrative should be more credible than the disinformation that is to be debunked.^37^ This entails deconstructing anti-democratic narratives, cultivating an informed debate and building one’s own narratives on truth, values and vision. In the following section, we present our case study. After a short presentation of the case itself, we attempt to determine the degree of trust, social capital, and a credible narrative. We thus expect to obtain a picture of the resilience of Dutch society, which, again, affects the political determination of the country and ultimately its contribution to deterrence.

## The Case

On 17 July 2014, flight MH17 was shot down over Ukraine. In the hours following the crash, different narratives began to emerge: Western media claimed that pro-Russian separatists downed the aircraft, while the Russian government blamed the Ukrainian military.^38^ Furthermore, the Russian government stated that no missile had crossed from Russia into Ukraine. On 21 July, the United Nations Security Council unanimously adopted Resolution 2166, which condemned the shooting, called for an independent international investigation, and urged all UN member states to cooperate fully. Shortly after the crash, Dutch prime minister Mark Rutte began to use a triple narrative: bring the victims home, discover what happened, and find those responsible. On 18 July, he declared that he would not rest until the perpetrators were brought to court.

In October 2015, the Dutch Safety Board (DSB) issued its final report on the crash, concluding that a BUK surface-to-air system shot down the aircraft. In September 2016, the Dutch-led joint investigation team (JIT), which included police and judicial authorities from Australia, Belgium, Malaysia, the Netherlands, and Ukraine, presented its findings. The JIT disclosed that a missile was fired from an area that pro-Russian separatists controlled. In addition, the JIT found that the BUK was transported into Ukraine from the Russian 53rd Anti-Aircraft Brigade, based in Kursk. After shooting down flight MH17 and its passengers, the BUK returned to Russia.^39^

In May 2019, *De Groene Amsterdammer* reported that in the first two days following the crash, a St. Petersburg–based Russian troll factory issued at least 65,000 tweets blaming Ukraine for the shooting. Most of them were in Russian.^40^ In the aftermath of the crash, alternative theories emerged, inspired predominantly by events undergoing investigation and public prosecution.^41^ The first theory was that a Ukrainian Sukhoi Su-25 jet fighter downed the aircraft. The deputy chief of staff of the Russian Armed Forces, General Andrej Kartapolov, endorsed this theory in a press conference on 21 July 2014. Two days earlier, “Carlos”, a so-called Spanish air traffic controller based in Ukraine, had initiated this theory over several tweets. This Twitter account proved to be fake.^42^ The state-sponsored media outlet Russia Today even executed a test with a Su-25 to prove that the aircraft could reach the same altitude as flight MH17.^43^

Russian governmental institutions seem to propagate disinformation regardless of the consequences for their reputations. Disinformation activities originated primarily from actors in the Russian Federation but were also disseminated from Ukraine. The Security Service of Ukraine (SBU)^44^ disclosed via Interfax that it had prevented a shrewd Russian attack on Dutch Foreign Minister Bert Koenders while he was visiting Ukraine.^45^ Conversely, in August 2014, the SBU had propagated in a press conference that the target had not been flight MH17 but Aeroflot flight 2074; the intent had allegedly been to create a *casus belli* for the Russian Federation to invade Ukraine. The SBU and other Ukrainian government institutions quickly abandoned this theory.^46^ Even in the Netherlands, an instance of disinformation involving a Dutch member of Parliament from the Christian Democratic Party occurred: He provided a fake witness with a text he had prepared.^47^ Later, the party ended his role as spokesperson in the case of flight MH17.^48^

During the months and years to follow, the Russian narrative stabilized around the dominant message that Ukraine is responsible and all investigations are biased to discredit the Russian Federation. As soon as evidence that a surface-to-air missile caused the catastrophe emerged, the narrative changed from a Ukrainian Su-25 fighter jet to a Ukrainian BUK missile having been launched from Ukrainian-held territory. Russian state-sponsored outlets such as Russia Today and Sputnik supported both narratives. In the following sections, we monitor how Dutch society demonstrated societal resilience in the face of disinformation activities and attempts to sow doubt and discredit the investigation into the disaster. We focus on trust, social capital, and the narratives used in the discourse that surfaced after the disaster.

## Trust

Trust is the willingness of citizens to believe in the authorities’ ability to manage a crisis. All interviewees who participated in this research mentioned this factor. For instance, the anonymous respondent from the government’s crisis management organization asserted: “We were as open as we possibly could be towards the relatives of the victims and showed them what we were doing. This created mutual understanding and trust.” The DSB acted similarly; Wim van der Weegen, as head of administrative affairs, advice, and communications/spokesperson, stated: “We practised openness as long as possible.” The authorities understood the importance of exercising trust to avoid confusion and thus enhanced societal resilience against disinformation.

**Fig. 19.1:**
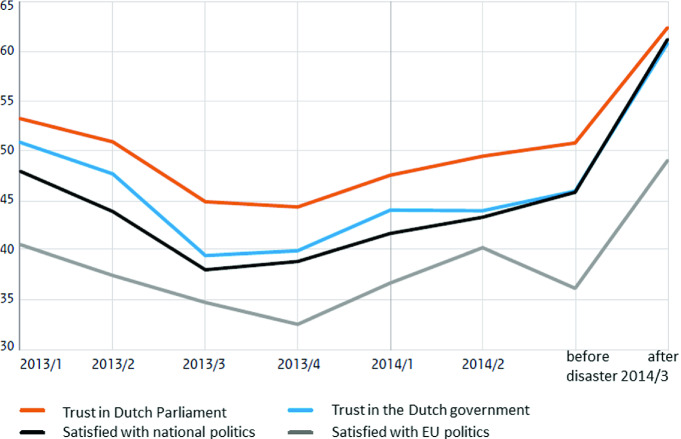
Political confidence increases after the disaster (*Source* Netherlands Institute for Social Research (SCP) 2014_3, p. 2.)

The Sociaal Cultureel Planbureau monitors social developments in Dutch society. Shortly after the shooting down of flight MH17, this institution reported that trust in the government had risen from 46 to 61% (see Fig. 19.1).^49^ The September 2018 report indicated that 56% of the Dutch trust the government, which is a common figure in the Netherlands.^50^ In fact, the Dutch demonstrate a stable trust level of over 70% in their legal system.^51^ Research by the Centraal Bureau voor de Statistiek revealed a similar outcome between 2012 and 2018.^52^

The respondent from the government’s crisis management organization had the same thoughts: “I think the Dutch population is still confident about the approach. They have faith we do this in a proper way and conscientiously.” Public trust in the government is a clear sign of societal resilience, for trust is the opposition of doubt. As the government’s crisis management organization’s source mentioned: “In the first days, there was a lot of criticism towards the government’s approach, mainly because of its being too careful. This really changed as soon as we started to repatriate the victims. Suddenly, we received a lot of positive feedback.”

Whenever the DSB or JIT publicized a report, Russian sources responded with messages to undermine trust in these institutions. The government’s crisis management organization confirmed: “The Dutch minister of foreign affairs summoned the Russian ambassador … to make clear Russia had to stop with its continuous efforts to discredit the DSB and JIT investigations. This step was taken when high-ranking Russian officials had started to amplify this narrative.” Gaining and maintaining the trust of the victims’ relatives has been a key factor in facilitating the investigation processes. As Piet Ploeg, chairman of the MH17 Disaster Foundation (see below) asserted: “Whatever these [Russian] people did to convince us, they simply didn’t succeed because the vast majority of the relatives had faith in the government, the Public Prosecution Service, and the Dutch Safety Board. Even the more activist part of the relatives did.” Ploeg thus clarified the correlation between trust and societal resilience against disinformation.

When one considers trust in a societal context, it is necessary to seek trust in social media as well, since social media platforms are also used for disseminating disinformation. Again, *De Groene Amsterdammer* analyzed over 65,000 tweets concerning flight MH17 that a Russian troll factory had sent in July 2014.^53^ While the use of social media could not be researched extensively due to the time and space limitations of this chapter, it is clear that social media platforms have had a muting effect on the debate. Trust in the Dutch authorities was a constant factor. Like in other crises, such as the recent outbreak of COVID-19, trustworthy leadership guides the nation. Shortly after the disaster of flight MH17, trust in the Dutch government rose to 61%; afterward, trust returned to normal levels, regardless of disinformation activities (see Table 19.1).

**Table 19.1:** Opinions on the Dutch government’s actions in the case of flight MH17 (*Source* Peil.nl 2019)

What is your opinion on the government’s actions in this case over the past year?	8-2-2015 (%)	5-27-2018 (%)
Positive	14	11
Quite positive	31	24
Neutral	26	30
Negative	13	13
Quite negative	15	16
Don’t know/no opinion	1	6
Total	100	100

## Social Capital

The Russian narratives have been particularly hurtful to the families and friends of the 298 victims. Shortly after the crash, the MH17 Disaster Foundation was founded to assist and support the victims’ families. The PPS established a team of trained family counsellors to assist the families and retrieve the personal belongings of their loved ones for identification purposes. Its chairman, Piet Ploeg, attributes a key role to these counsellors: “An effect of the family counsellors was that they kept us together.” This unity has made it difficult to create divisions within this group. National symbols have also encouraged unity. As Ploeg stated: “The government and the royal family were very much involved. This was very supportive for the victims of the relatives.” This commitment, as demonstrated by the government’s extensive support to the families and the role of national symbols, as signified by the Dutch royal family’s involvement, proves how social capital in the case of flight MH17 contributes to societal resilience against the effects of disinformation. In this case, social capital helped to strengthen the ties between the disorientated and otherwise affected families and the government managing the emerging crisis.

Fons Lambie, an RTL journalist, mentioned how social capital contributes to societal resilience. He described the long line of funeral vehicles that transported the bodies from Eindhoven Airport to Hilversum over the Dutch highways. Standing on bridges and flyovers, thousands of people offered their respect to the funeral procession. Lambie: “This massive expression of grief that paid tribute to the hearse columns when the bodies returned really united the nation and made it very hard to cause divisions.” However, according to Lambie, these circumstances could also change: “What would happen if a populist prime minister, like Thierry Baudet, would take office?” This event could very well cause a shift in thinking about flight MH17 as populist authoritarian movements in the Netherlands strive to improve relationships with Russia.^54^ These parties also support the Russian narrative, as illustrated in a poll published on 27 May 2018 (see Table 19.2). It is mainly among the constituencies of the populist Party for Freedom and Forum for Democracy that sympathies for the Russian narratives can be found.^55^

**Table 19.2:** Opinion poll 27 May 2018: Who is responsible for the shooting down of flight MH17? (*Source* Peil.nl 2019)

Which party in the conflict in Ukraine shot down the aircraft?	5-27-2018 (%)
The Ukrainian army	5
Separatists who strive to separate Eastern Ukraine	22
The Russian army	52
Don’t know/no opinion	21
Total	100

On 9 March 2020, the official trial against Igor G., Sergey D., Oleg P., and Leonid K.—three Russians and one Ukrainian from Eastern Ukraine—for the murder of the 298 passengers on the Malaysian Airlines jet began in a court near Schiphol Airport. As two-thirds of the victims were Dutch and a Dutch team conducted the investigation, the trial is being held in the Netherlands. The public trial serves to deter by delegitimization as every single detail disclosed will discredit the alternative narratives that Russian actors have issued.

From day one, the Dutch authorities have made significant efforts to support the families of the victims. Despite the attempts to create division, overall, these families kept the rows closed with the crime investigation teams.

## Credible Narrative

In the case of flight MH17, narratives have played a critical role. In fact, the case can be characterized as a battle of narratives. From the beginning, official and state-sponsored media outlets from Russia have blamed Ukraine for the shooting down of flight MH17. As soon as evidence began to collect, these media turned to a second, more defensive approach that strove to discredit the DSB and the findings of the JIT. The Dutch government’s narrative has consistently focused on three courses of action: bringing the victims home, investigating the tragic crash, and finding those responsible. The respondent from the government’s crisis management organization stated: “Prime minister Rutte uses this frame over and over to explain why things take so much time and uses it to show compassion with the relatives of the victims.” As Piet Ploeg confirmed: “The government had a narrative as clear as a three-stage rocket: get back the victims, find out what happened, and bring the perpetrators to court.”

The narrative of the Dutch government proved to be a helpful frame to counter disinformation since each of the respondents recognized the threefold approach. The design was well chosen to maintain a distinct separation in responsibilities between governmental institutions. It was also propagated as a frame for all separate activities. The government’s crisis management organization’s source commented: “In all those years, the prime minister always has been very clear about the different independent roles of the institutions DSB and JIT because he has been very much aware of the risk he would take to be framed as biased by the Russians.” The design and execution of this narrative contributed to societal resilience because it served as a frame to counter disinformation.

Van der Weegen (DSB) offered a prime example of how creating an image can vigorously enhance resilience against disinformation. He stated: “The (iconic) reconstructed hull image has been thoroughly considered and designed in support of DSB’s narrative (i.e., the findings to be presented cogently in a threefold approach: report, computer animation, and presentation in front of the reconstructed cockpit).”

The Dutch government communicated a triple narrative of returning the victims home, establishing what happened, and bringing the perpetrators to justice. The agenda of the Russian Federation was clear to all involved, and they responded by being aware of disinformation, shielding information from cyberattacks, and avoiding mistakes that could fuel disinformation activities.

## Conclusion

In this chapter, we tried to establish is how subversive Russian activities were taking place and what measures were taken by the Netherlands government in order to counteract them. We monitored societal resilience by looking for the presence of trust, social capital, and credible narratives in reaction to disinformation activities after a disruptive event. All these elements appeared to be present in the MH17 case. In Dutch society, a feeling of trust in the government, the PPS, and the DSB emerged. Despite concerns about pro-Russian populist parties, social capital proved to be relevant to strengthening the ties between the disoriented and otherwise affected families and the government managing the crisis. The narrative that the government used—bring the victims home, discover what happened, and bring the perpetrators to court—was a robust frame in countering the effects of disinformation on Dutch society. Russian narratives were discredited. An independent and transparent criminal procedure is underway; it is aimed at truth-finding and fine-tuned toward the individual perpetrators and therefore an antidote to disinformation. To illustrate whether that contributes to deterrence, we have divided deterrence into deterrence by denial, punishment, and delegitimization.

Overall, the handling of the MH17 case has reinforced deterrence by denial. Russian disinformation has thus far proven to be unconvincing for most Dutch target audiences. Moreover, the prime minister has demonstrated his commitment to the case, while the Dutch authorities have upheld a consistent narrative and fostered trust and social capital by example. The government has also respected the independent position of others, such as the PPS and the free press, in their search for the truth. Other sources of information, such as free independent news networks and digital forensic networks, have been paramount in discrediting disinformation and allowing the public to conclude what it actually is: lying. In the case of flight MH17, one newspaper disclosed how a Dutch MP instructed a fake witness in a meeting, and a Dutch weekly disclosed how a well-known Russian troll factory based in St. Petersburg disseminated over 65,000 tweets shortly after the crash. Digital forensic platforms such as Bellingcat and the EEAS initiative of the EU pose a serious threat to the originators of disinformation. During the prosecution process, for instance, civic journalists were a great help in disclosing the exact route of the BUK installation entering and leaving Eastern Ukraine. In summary, insofar as Russian alternative narratives have not been able to gain any real foothold in Dutch society, deterrence by denial has been enhanced.

The criminal proceedings against the suspected perpetrators of the shooting down of flight MH17 are themselves a movement toward punishment. The DSB and the Public Prosecution Service have played a key role in unravelling the catastrophic events, as well as prosecuting the alleged perpetrators. The latter formed the core of the international JIT; the research was thorough, transparent, and followed a fixed protocol. Furthermore, the DSB and JIT were aware of the risk of disinformation and introduced additional checks and balances to avoid mistakes. A resolution of the Security Council of the United Nations supported the entire procedure.

Finally, the legal proceedings and the MH17 trial, which began on 9 March 2020, have contributed to deterrence by delegitimization. The trial has demonstrated not only the determination of the Dutch to bring the perpetrators to court but also that every single detail that surfaces will discredit the alternative facts and narratives that Russian sources have disseminated. We have taken the killing of the 298 passengers on flight MH17 as our case. What we observed was best described by the Dutch government official who remarked: “I think the Dutch population is still confident about the approach. They have faith we do this in a proper way and conscientiously.” The case of flight MH17 offers a prime example of how a resilient society can deter an actor from conducting effective disruptive campaigns.

## Footnotes

1 Rietjens 2019.

2 Galeotti 2017.

3 Van Doorn 2019.

4 They are members of the Dutch government, the Dutch Public Prosecution Service (PPS), the Dutch Safety Board (DSB), the Dutch press and relatives of the victims.

5 Van Doorn 2019; a significant research element of Van Doorn’s master thesis consisted of a process tracing approach of disinformation activities to gain insight into the relationship between flight MH17–related events and debunked cases of disinformation. This information was ordered into a comprehensive timeline. Due to space limitations, we could not incorporate this timeline here, but it can be found here: https://www.linkedin.com/posts/ceesvandoorn_extensive-timeline-of-disinformation-in-the-activity-6668960213137154048-lzhN.

6 This phenomenon can be seen in other cases as well, such as the war in Syria and the poisoning of Sergei and Julia Skripal in March 2018. An accessible digital collection was created for all those who have dispersed disinformation. The alleged meddling in the 2016 U.S. presidential elections has raised the awareness of disinformation and urged governments, including the Dutch, to act. The Dutch government has acted strategically (i.e., in policy documents) as well as practically, as in the case of an awareness campaign against fake news that the Ministry of the Interior launched in March 2019.

7 Wilner 2011, pp. 4–5.

8 MCDC Countering Hybrid Warfare Project 2019, p. 3.

9 Cullen and Reichborn-Kjennerund 2017, p. 8; see also Davis 2015, p. 5.

10 Strachan n.d., p. 52.

11 Smith 2005, p. 6.

12 Davis 2015, p. 21.

13 Galeotti 2017, pp. 5–6.

14 Treverton et al. 2018, pp. 9, 43, 52.

15 AIVD 2020, p. 81.

16 Brief ongewenste buitenlandse inmenging 2018, p. 1.

17 Galeotti 2017, p. 6; Nicolini and Janda 2016, p. 82.

18 Smith 2005, p. 6.

19 Rühle 2015.

20 Daalder 2017, p. 38.

21 Wilner 2011, pp. 26–27.

22 Knopf n.d., p. 18.

23 MCDC Countering Hybrid Warfare Project 2019, p. 3.

24 Rodin 2014, p. 3.

25 Rodin 2015, p. 63.

26 Fjäder n.d., pp. 122–123.

27 Coaffee and Wood n.d., p. 505.

28 Giegerich 2016, p. 65.

29 Galeotti 2017, p. 15.

30 Gray n.d., p. 258.

31 Whither n.d., p. 65.

32 Nicolini and Janda 2016, p. 83.

33 Statewatch 2015, p. 22.

34 Stockholm Resilience Centre 2015; Noordegraaf et al. 2018; Versteegden 2018.

35 Versteegden 2018, p. 26.

36 Durodié 2005, pp. 42–44.

37 Versteegden 2018, p. 26.

38 Noordaa and Van der Ven, *Nepnieuws uit Sint Petersburg*.

39 *Update investigation JIT MH17—press meeting*.

40 Van der Noordaa and Van der Ven 2019.

41 Rudin 2016.

42 Luhn 2014.

43 Rudin 2016.

44 SBU stands for Sloezjba Bezpeky Oekrajiny.

45 RTL Nieuws 2014.

46 Toler 2018a, b.

47 Van der Peet 2017.

48 Van Ast 2017.

49 Netherlands Institute for Social Research (SCP) 2018_3.

50 Netherlands Institute for Social Research (SCP) 2018_3, p 15.

51 Ibidem, Fig. 1.6.

52 CBS 2019.

53 Van der Noordaa and Van der Ven 2019.

54 The Hague Centre for Strategic Studies 2017, p. 88.

55 Peil.nl 2018.
